# The contribution of recombination to heterozygosity differs among plant evolutionary lineages and life-forms

**DOI:** 10.1186/1471-2148-10-22

**Published:** 2010-01-25

**Authors:** Juan P Jaramillo-Correa, Miguel Verdú, Santiago C González-Martínez

**Affiliations:** 1 Departamento de Sistemas y Recursos Forestales, Centro de Investigación Forestal, CIFOR-INIA, Carretera de La Coruña, km 7.5, ES-28040 Madrid, Spain; 2 CIDE, Centro de Investigaciones sobre Desertificación (CSIC-UV-GV). Camí de la Marjal s/n Apartado Oficial. ES-46470 Albal, València, Spain; 3 Departamento de Ecología Evolutiva, Instituto de Ecología, Universidad Nacional Autónoma de México, Ciudad Universitaria, Tercer circuito Exterior, Apartado Postal 70-275, México, DF

## Abstract

**Background:**

Despite its role as a generator of haplotypic variation, little is known about how the rates of recombination evolve across taxa. Recombination is a very labile force, susceptible to evolutionary and life trait related processes, which have also been correlated with general levels of genetic diversity. For example, in plants, it has been shown that long-lived outcrossing taxa, such as trees, have higher heterozygosity (*H*_e_) at SSRs and allozymes than selfing or annual species. However, some of these tree taxa have surprisingly low levels of nucleotide diversity at the DNA sequence level, which points to recombination as a potential generator of genetic diversity in these organisms. In this study, we examine how genome-wide and within-gene rates of recombination evolve across plant taxa, determine whether such rates are influenced by the life-form adopted by species, and evaluate if higher genome-wide rates of recombination translate into higher *H*_e _values, especially in trees.

**Results:**

Estimates of genome-wide (cM/Mb) recombination rates from 81 higher plants showed a significant phylogenetic signal. The use of different comparative phylogenetic models demonstrated that there is a positive correlation between recombination rate and *H*_e _(0.83 ± 0.29), and that trees have higher rates of genome-wide recombination than short-lived herbs and shrubs. A significant taxonomic component was further made evident by our models, as conifers exhibited lower recombination rates than angiosperms. This trend was also found at the within-gene level.

**Conclusions:**

Altogether, our results illustrate how both common ancestry and life-history traits have to be taken into account for understanding the evolution of genetic diversity and genomic rates of recombination across plant species, and highlight the relevance of species life forms to explain general levels of diversity and recombination.

## Background

Recombination, the re-assortment of genetic variation into novel haplotypic arrangements by both homologous crossover and gene conversion [1], is one of the main sources of genetic diversity in Eukaryotes. It decouples neutral variation from linked deleterious mutations that are consistently eliminated by selection, and from beneficial variants, which would tend to be fixed [2]. Recombination can potentially increase haplotype variation and expected heterozygosity (*H*_e_) [3], either directly (for instance, if mutagenic) or indirectly (through the effects of selection). Thus, a higher recombination rate should translate in higher genetic diversity within a given genomic region, population or even species.

At the within species level, recent evidence from DNA sequence analyses has shown that recombination might be as, if not more, frequent as mutation (e.g. [3] in wild barley, [4] in Scots pine). These observations also hint that there could be a positive correlation between the rate of recombination and *H*_e_. Although such as association is not always straightforward due to the labile nature of recombination and its susceptibility to selective, stochastic and life trait related processes [e.g. [5-8]], a direct and positive correlation between the rate of recombination and heterozygosity is expected under recurrent background selection regimes [9,10]. Indeed, such a correlation has been observed in both animals and plants [e.g. [11-15]], although balancing selection, selective sweeps and/or higher mutation than recombination rates [e.g. [16,17]] could have the capability to blur it within a few generations.

Across species, little is known about how the rate of recombination evolves or how it is correlated with the average levels of genetic diversity. In mammals, the comparison of orthologous gene regions within and across species has shown that a shared evolutionary history is a poor predictor of the rate of recombination [18], which suggests that such a rate evolves at a rather fast pace at short scales within the genome. However, this pattern does not seem to be extended at the average or genome-wide level, as the rates of recombination, measured from genetic maps, showed a strong phylogenetic signal, with more closely related species having more similar recombination rates [19]. Such results might be due to the fact that broad-scale recombination rates are constrained by meiotic mechanisms during the disjunction of homologous chromosomes [e.g. [20,21]]. As a consequence, in mammals, the rate of evolution of the genome-wide rate of recombination could be much slower [19]. Such information is still missing in plants, although a similar correlation between species relatedness and genome-wide recombination rate could be expected given the fast speciation rates of some lineages, especially angiosperms, and the fact that the disjunction of plant homologous chromosomes is ruled by similar constraints that in animals [20].

The average levels of *H*_e _are, on the other hand, expected to change quickly and on short evolutionary time scales due to their sensibility to stochastic forces [22]. Among higher plants, the widespread, outcrossing and perennial taxa have consistently higher *H*_e _at allozymes and SSRs than their endemic, selfing or annual counterparts, independently of any possible phylogenetic relationship [22,23]. Nevertheless, sequencing approaches in trees, most of them widespread, outcrossing and perennial, have surprisingly shown that these taxa, in spite of their high average heterozygosities, bear relatively low levels of nucleotide diversity at the DNA sequence level when compared to plants with different growth habits [reviewed by [24]]. These results could be the product of a phylogenetic artefact, given that most of the trees studied so far belong to particular clades (e.g. conifers, *Populus*). However, this apparent contradiction could also suggest that recombination, instead of mutation, might be more involved in maintaining or generating the high levels of *H*_e _observed in trees, than in shrubs or herbs.

In this context, it follows then that three hypotheses are worth testing: (i) whether the genome-wide rate of recombination of higher plants shows a phylogenetic signal; (ii) whether these rates differ between species life-form (tree, shrub or herb), with trees having higher rates of recombination than other plant life-forms; and (iii) whether higher rates of genome-wide recombination translate into higher levels of *H*_e_. In the present study, we addressed these three key issues on higher plants by using a comparative phylogenetic approach on a large sample of average rates of recombination, estimated from total genetic map lengths and physical genome sizes, and mean values of *H*_e_, calculated with SSR loci. We provide a first insight into the evolution of the genome-wide recombination rate across plant lineages, and show how this source of genetic variation is affected by different life traits once the phylogenetic signals of all parameters are accounted for. The control of such signals allowed us to discern whether species share similar levels of recombination due to common ancestry and/or to convergent life-history traits, such as growth habit, that have arisen independently in different lineages [e.g. [25-27]]. Finally, we made a preliminary survey on the plant nucleotide sequences available on public databases, in order to determine if the trends observed across species at the genome level can also be observed at the within-gene scale.

## Results

**Figure 1:**
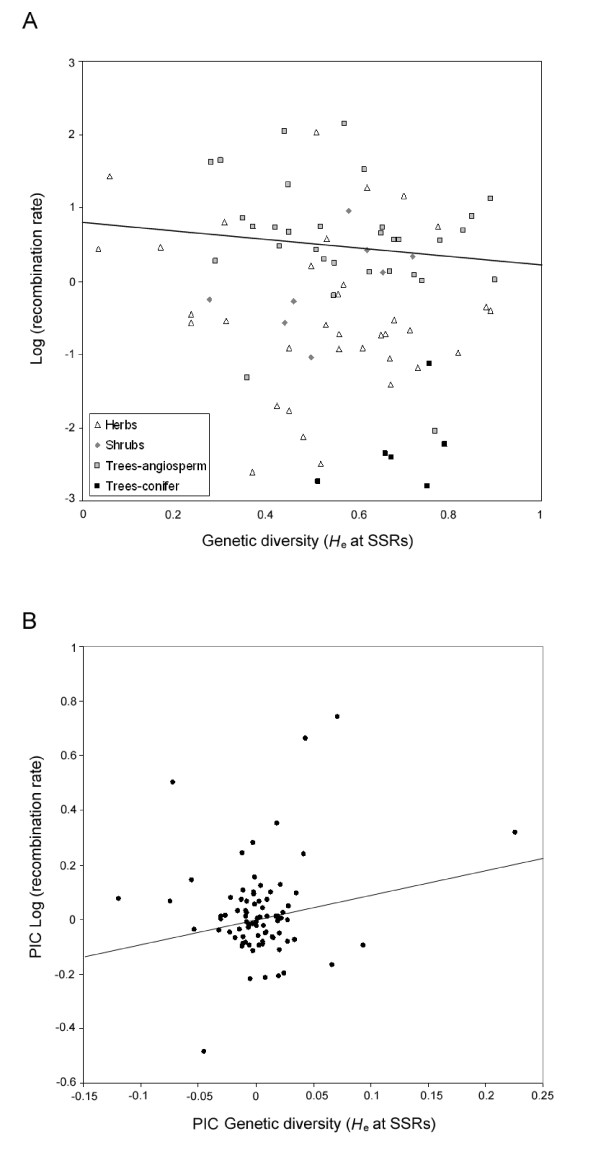
**Correlation between the genome-wide rate of recombination and *H*_e _in 81 higher plants species**. Genome-wide rate of recombination decreases with *H*_e _when the phylogenetic relationships of species are not taken into account (A), but increases when these relationships are accounted for by means of phylogenetic independent contrasts (PICs) (B). In box A, each species has been labelled according to its life-form (herb, shrub, angiosperm tree or conifer tree).

**Figure 2:**
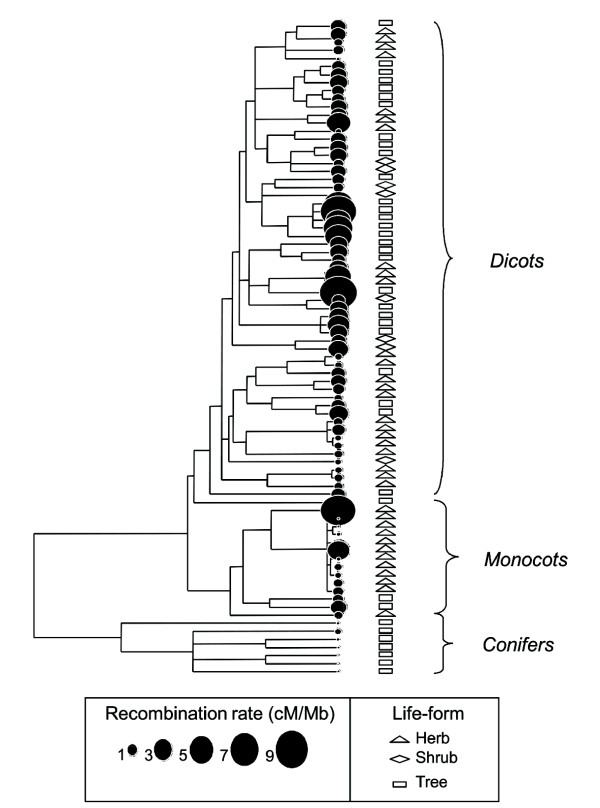
**Phylogenetic distribution of the genome-wide rate of recombination for 81 higher plant species classified according to their life-form**. Dot sizes are proportional to the recombination rate following the scale shown below the tree. The life-form of each species is indicated by rectangles (trees), diamonds (shrubs) or triangles (herbs) in front of each clade.

Estimates of genome-wide rate of recombination and *H*_e _at SSR loci were gathered for 81 higher plant species (i.e. dicots, monocots and conifers) that were classified according to their type of life-form (tree, shrub or herb). A preliminary standard correlation analysis (i.e. uncorrected for the phylogenetic relationships among species) revealed that these two traits were negatively correlated, and that trees had higher recombination rates than herbs but similar to shrubs (Table 1). However, the examination of the phylogenetic distribution of our data suggested that closely related species, such as conifers, tended to have similar rates of recombination, as revealed by their close location at the bottom-right corner of Fig. 1a, and by an analysis of residuals (not shown). Such a trend was made further evident after mapping the rates of recombination of our 81 species in a phylogenetic tree (Fig. 2). A set of standardized phylogenetically independent contrasts (PICs) made at the tips of this tree revealed a significant phylogenetic signal for this trait (*K *= 0.35; *P *< 0.001), as the observed variance of the PICs for the recombination rate (0.0433) was much lower than expected by chance (0.2469).

**Table 1:** Different generalized linear models (GLMs) showing the relationship between the genome-wide rate of recombination (log-transformed), the expected heterozygosity (*H*_e_) at SSRs and the life-form of 81 higher plant species.

	Uncorrected model^a^			Phylogeny-corrected model #1^a^			Phylogeny-corrected model #2^b^		
			
	Estimate	S.E	*p-value*	Estimate	S.E	*p-value*	Estimate	S.E	*p-value*
Intercept	0.842	0.451	0.066	-1.527	0.999	0.150	-0.135	0.729	0.856
Diversity	-1.206	0.691	0.085	0.836	0.296	0.014	0.847	0.149	9.5 × 10^-5^
									
Life form									
Herbs	-0.645	0.275	0.021	-0.858	0.334	0.023	-0.892	0.168	1.8 × 10^-4^
Shrubs	-0.235	0.451	0.604	-0.726	0.396	0.090	-0.744	0.201	2.8 × 10^-3^
Conifer trees	---	---	---	---	---	---	-2.624	0.993	2.2 × 10^-2^

^a ^In these two models, all trees (conifers and angiosperms) were considered as a single life-form, which was fixed as baseline for the contrasts.

^b ^In this model, the conifer and angiosperm trees were considered as separate "life-forms". The angiosperm trees were fixed as baseline for the contrasts.

**Figure 3:**
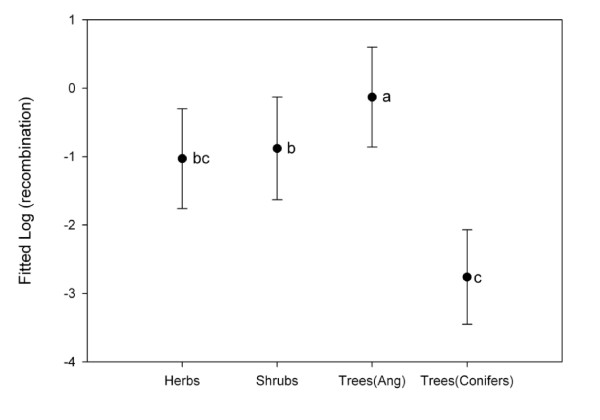
**Estimates of (log) genome-wide rate of recombination fitted to a phylogeny-corrected model for 81 higher plant species**. Bars represent ± 1 S.E. confidence intervals. Species were classified according to their life-form (herbs, shrubs or trees) and considering angiosperm (Ang) and conifer trees separately. Significant differences in the logarithm of recombination rates between life forms are indicated with different letters.

Following these results, a new GLM model was built by taking the phylogenetic relationships of species into account. This model revealed a positive and significant correlation (0.83 ± 0.29) between the genome-wide rate of recombination and *H*_e _(Fig. 1b), and showed that life-form explained a significant portion of the variation found in the rate of recombination across taxa (Table 1). The individual coefficients estimated for each particular trait integrated into this model further revealed that the rate of recombination in trees was significantly higher (-1.52 ± 0.99) than in herbs (-2.38 ± 1.03; *P *= 0.023), and marginally superior than in shrubs (-2.25 ± 1.06; *P *= 0.090; Fig. 3).

In order to avoid any potential bias due to the unusually large genome size of conifers (see ref. [28] and Additional file 1), a second phylogeny-corrected model was built after separating these taxa from the angiosperm trees. This model also revealed a positive and significant correlation between the genome-wide rate of recombination and *H*_e_, with a very similar value to the one obtained with the previous model (0.84 ± 0.22). Furthermore, an important effect of life form, with conifer trees having significantly lower recombination rates than angiosperm trees and shrubs (Table 1, Fig. 3), was also observed with this model.

Finally, in order to determine if the trends observed at the genome-wide level could also be inferred at a finer scale (i.e. within the gene space), a comparative analysis was performed on within-gene recombination rates recalculated from available nuclear gene DNA sequences retrieved from public databases (see Additional file 2). Briefly, only DNA sequences from single-copy nuclear genes spanning at least 800 base pairs (bp), having a minimum of 10 segregating sites, and sampled for more than 20 chromosomes were taken into account. Shorter sequences, sequences that were obtained from diploid, and thus unphased material, or sequences obtained from only a few (i.e. less than 20) individuals were deliberately excluded. This reduced dramatically the sample size of the survey, but assured us the possibility of calculating the most accurate recombination rates possible (see Materials & Methods for more details).

The patterns obtained roughly point in the same direction than the trends observed at the genome-wide level (Kruskal-Wallis test; *P *< 0.01; see Additional file 2). All the within-gene recombination rate estimates (i.e. *Rm*, *ρ*_MC_, *ρ*_T05_, *ρ*_MC_/*θ*_MC _and *ρ*_T05_/*θ*_T05_) were lower in conifers than in angiosperms, while most of the non-conifer trees exhibited higher values than the non-domesticated herbs and shrubs, with the possible exception of *Zea mays *ssp. *parviglumis*. These results are obviously only exploratory, but they do open the door for extended comparisons once enough genomic data and physical genetic maps are available.

## Discussion

The different models implemented in this study provide evidence that the genome-wide rate of recombination evolves slowly across higher plant lineages, with phylogenetically close species having more similar rates than distantly related taxa. In addition, once phylogenetic relatedness is accounted for, a positive and significant correlation between the average rate of recombination and *H*_e _was observed, with life-form explaining a substantial part of the differences observed across taxa. However, the significant taxonomic component made evident by our models, particularly when the conifer trees were considered separately from their angiosperm counterparts, suggests that additional ancestral evolutionary features are also playing a key role modelling both *H*_e _and the genome-wide rate of recombination, especially in long-lived taxa such as forest trees.

### Phylogenetic signal of plant genome-wide rate of recombination

The phylogenetically independent contrasts performed herein demonstrate that the average rate of recombination is a relatively well conserved trait among closely related plant lineages. Both the large number of species (81) included, and the use of randomised datasets to determine significance, provided enough power to detect the presence (or absence) of a phylogenetic signal in our recombination rate data. The distribution of values was clearly non-random (Figs. 1a &2), which suggests that the genome-wide rate of recombination of one species could be used to predict the same measure in related taxa for which no genetic map is still available [19]. Such a possibility is reinforced by the high levels of synteny and macro-colinearity observed in comparative genetic mapping surveys among congeneric taxa (e.g. [29] in the Rosacea, [30] in conifers). However, particular issues related to the selective or stochastic forces that shape independently each particular species might tend to blur these predictions. Indeed, different ecological and selective patterns might result in altered levels of recombination [6-8]. For example, domesticated plants tend to have higher rates of recombination than their wild ancestors or relatives [7]. Nevertheless, our comparative analyses pointed out that the putative differences between the genome-wide recombination rates of domesticated taxa and their undomesticated relatives were low when they were compared to the differences observed between distantly related species (see Materials & Methods for more details).

### Plant life-form and genome-wide rate of recombination

The simultaneous presence of high *H*_e _estimates at allozymes and SSRs [22,23] and high genome-wide rates of recombination observed in trees when compared to other plant life-forms, suggests that recombination might be playing a relevant role in generating genetic diversity in these taxa. Common biological traits of trees, such as their large population sizes, extensive gene flow, outcrossing mating systems and long generation times, point to common evolutionary forces that might be shaping their amounts of genetic diversity in a similar way [22,31]. These common traits have been often invoked to explain the differences observed in substitution and diversification rates between woody angiosperm lineages and their herbaceous counterparts [e.g. [25-27]]. Further tree life-history features, such as their higher basic number of chromosomes, have also hinted that they might have higher genome-wide recombination rates than herbs or shrubs [32,33]. This factor is expected to promote diversity through its direct impact on the number of crossing-overs and thus, in the rate of genome-wide recombination. However, previous works have shown, by correlating the number of chiasmata per bivalent with different plant biological traits, that perennial and outcrossing angiosperms (including trees) had lower recombination rates than their annual or selfing counterparts [7]. The contradiction between these findings and our results might be explained by the important contribution of gene conversion to the mean rate of recombination in higher plants. Such a contribution (as estimated by *f*, the ratio of gene conversion to cross-over) spans between 0.5 and 14 [e.g. [2,34,35]], and it is not comprised in the direct count of chiasmata, while it is included in the recombination rates derived from total genetic map lengths [19], such as those estimated herein. However, for this to be true, it is necessary that the rate of gene conversion varies systematically between perennials-outcrossers and annual-selfers. Although so far there is no evidence for such a difference, it is expected that species with higher average *H*_e_, such as forest trees, will exhibit higher rates of gene conversion because gene conversion can only be detected in heterozygous sites [3,35,36]. In any case, the growing number of surveys estimating the contribution of gene conversion to recombination should eventually allow testing for such eventual differences between trees and other plant life-forms.

### Correlation between recombination rates and heterozygosity

The contribution of recombination to genetic diversity, especially *H*_e_, and the putative correlation of these two factors has received increasing attention in the recent years. Various theoretical works predict that, within a genome, there should be a positive correlation between the rate of recombination and genetic diversity at neutral loci under different regimes such as common selective sweeps, genetic hitchhiking combined with low mutation rates and/or background selection [9,10]. Such a correlation has been indeed observed in different plant and animal taxa [e.g. [11-15]]. However, such regimes would hardly explain the correlation observed herein across higher plants, unless the same selective forces were acting in the same direction and determining, in the very same way, the genetic variability across closely related species. An alternative explanation would be that differences in *Ne *or other life-trait related factors observed across taxa, such as gene flow or generation time length, were simultaneously affecting the rate of genome-wide recombination and *H*_e _[37]. On the other hand, several authors have remarked the mutagenic potential of recombination and its role in increasing nucleotide diversity [e.g. [2,38,39]]. For instance, an increased mutation rate has been observed during meiosis, and many of the newly detected mutations appeared to be correlated with neighbouring crossover events [38]. Such a correlation, if present across different species, might indeed explain the association observed herein between the average rate of recombination and *H*_e_. Moreover, if the mutation rate is indeed higher in regions with high recombination, then a correlation between recombination rate and heterozygosity could also be expected at a finer scale, for example among orthologous genes across species.

### Genome-wide vs. fine-scale recombination rates

Several studies have shown that the plant genome structure is highly heterogeneous and that recombination is not randomly distributed, occurring primarily within genes (reviewed by [2,40]). Such observation is reinforced by the similar gene-map lengths and the highly variable physical genome sizes reported for plant species (see Additional file 1), and thus raises the question of whether the trends observed herein for the genome-wide recombination rates can also be detected at a finer scale. Although an exhaustive analysis such as the one performed for the genome-wide estimates is out of scope for this study, and is probably still not possible due to the limited quantity of available data, this hypothesis was preliminarily tested by recalculating within-gene recombination rates on DNA sequences retrieved from public databases (see Additional file 2). Interestingly, trends were similar to those found for genome-wide recombination estimates, with conifers showing lower recombination than angiosperms and non-conifer trees (albeit very few data is available for this group) having higher values than herb and shrubs.

These similar levels of conservation in recombination rates inferred at different scales across plant species strongly differ from what has been reported for mammals. In these taxa, the rates of recombination at short scales appear to evolve faster than the rates at the genome-wide level [19], which suggest that different evolutionary forces might be operating at these scales. This opens two new questions that can be answered only tentatively for higher plants: at which scale is the rate of recombination evolving across-species? And, in consequence, what is the most evolutionary significant way of measuring recombination? If recombination occurs more often at the gene level, for example within gene hotspots, then the rates displayed in Additional file 2 should be the best way of measuring recombination. On the other hand, if there is a substantial portion of the total recombination events taking place at intergenic regions, and these events affect fitness, then the average genome recombination rates (Additional file 1) would be the most appropriate estimate. The answer to these questions is particularly important for understanding the evolution of conifers, which are in direct opposition to the general trend observed for angiosperms, where species with larger genomes have higher rates of recombination [7].

### Conifers *vs*. angiosperms

Among the surveyed tree species, conifers seemed to be a remarkable exception. Most of the genome-wide recombination estimates for these taxa were far lower than those from angiosperms (Figs. 1 &3; Additional file 1). Indeed, conifers were one of the clades that contributed the most to the differences observed between the non-phylogenetically and the phylogenetically-controlled models (Table 1 and Fig. 1). These differences suggest that the low rate of genome-wide recombination is an ancestral trait in conifers, and highlight the importance of considering phylogenetic relationships in comparative analyses such as those performed herein.

Different features of the conifer genome, like its large size, relatively small proportion of gene space and high amount of repetitive elements [28,41], can explain their low rates of genome-wide recombination. Previous genomic and sequencing initiatives have shown that conifers have a similar amount of genes, but within significantly larger genomes than angiosperms, a difference that is mainly due to a more ancient and substantial proliferation of repetitive and transposable elements [41]. In model plants (i.e. maize, rice and *Arabidopsis*), the genome regions where these elements occur have reduced levels of recombination [e.g. [2,40,41]], which hints that whole genomes rich in these repetitive and transposable elements, such as those from conifers, could have lower average recombination rates, such as it is shown in the present study. These elements have been previously associated with important structural and regulatory functions in model angiosperms [2,41], but their roles are still to be determined in other taxa.

The patterns exhibited by conifers, high levels of *H*_e _along with low amounts of nucleotide diversity at candidate genes (see [24] and Additional file 2) and low recombination rates at both the genome and within-gene scales, suggest that these species may have faced particular evolutionary forces that distinguish them from angiosperm trees. These forces could include frequent balancing selection and variation of mutation rates between coding genes and non-coding intergenic regions. On the other hand, it is also worth mentioning that some of the observed patterns could be due to imprecisions in the estimation of genome-wide recombination rates in conifers, prompted by the presence of large non-recombining regions or low gene density in large parts of the genome [e.g. [10]]. This would allow for high levels of *H*_e _in low recombination regions, which could be maintained by large ancestral population sizes and/or hybridization among related species [42,43], such as has been observed in *Arabidopsis lyrata *[44]. However, all these possibilities could only be explored once large genome-wide molecular datasets that include regions outside the gene space, pedigree surveys, and physical maps are available for a good number of conifers.

## Conclusions

Altogether, the results of the present study suggest that recombination is correlated with genetic diversity in higher plants, and that its effect is dependent on life-form, being more important in trees than in herbs or shrubs. This trend was observed at the genome-wide level, but could also hold at the within-gene scale. In addition, recombination not only appears to be conditioned by life-history traits, but also to rely on the evolutionary history of species, as shown by the differences observed between conifers and angiosperms at both genomic scales. These differences might by due to the proliferation of large amounts of non-recombining material, such as transposable elements, in the conifer genome.

## Methods

### Database assemblage

The average genome-wide recombination rate was calculated in cM/Mb for 81 plant species from 38 families including dicots, monocots and conifers. It was determined based on published estimates of total genetic map length and physical genome size as described elsewhere [19]. Diploid taxa were favoured and, whenever possible, domesticated species were joined by at least a wild relative of the same genus or family. After verifying that the differences between the recombination rates of domesticated plants and their wild relatives were not significant (*χ*^2^_9 _= 5, *P *= 0.83), we pooled all the data. Only those maps covering at least 60% of the genome were included in the database. Estimates of genetic map lengths (in cM) were corrected in order to account for variation in marker density across studies, and for undetected crossovers at distal terminal markers, as suggested elsewhere [19,45,46]. Estimates of physical genome size were either calculated from the haploid genome weights available at the Kew Plant C value Database [47], or retrieved directly from the primary literature. After classifying each species available according to its life-form (tree, shrub or herb), the estimates of mean *H*_e _at SSR markers were also collected. Microsatellites were preferred to other codominant markers due to their increasing availability in the literature, to their putative neutrality and to their association with non-repetitive DNA in plant genomes, including trees [48,49]. Only those *H*_e _values calculated from variation in at least five microsatellite repeats were included. Estimates based on population studies were favoured, but in some particular cases where such studies were not available (e.g. *Coffea canephora*, *Macadamia integrifolia*), values determined from preliminary screen panels had to be used. The complete database and references to the primary literature are available in Additional file 1.

### Phylogeny estimation, phylogenetic signal and evolutionary correlations

Species were assembled in a phylogenetic tree with the program Phylomatic as implemented in Phylocom 3.41 [50]. This program matched the genus and family names of our 81 taxa with those included in the megatree (R20050610.new) built by the Angiosperm Phylogeny Group [51]. The resulting phylogeny was calibrated using the age estimates from Wikstrom *et al*. [52] and adjusted by evenly distributing undated nodes between the nodes of known age [50].

The presence of a phylogenetic signal for the recombination rate was determined following the procedure of Blomberg *et al*. [53,54]. Briefly, the *K *statistic and its associated *P*-value were estimated from the variance of standardized contrasts, and compared with those obtained from a null model performed by reshuffling the trait values across the tips of the phylogeny. A significant phylogenetic signal was inferred at *α *= 0.05 when the mean observed variance of the contrasts was lower than 95% of the values produced by the null model. The phylogenetic independent contrasts were calculated for both the recombination rate and *H*_e _by using the APE package for R [55].

In order to determine the putative correlations between species ancestry, *H*_e _and life-form, three independent Generalized Linear Models were built. The first model was a non-phylogenetic (i.e. without taking the phylogenetic information of species into account) GLM with a Gaussian distribution of errors, which was made between the (log-transformed) recombination rate of species as dependent variable, and their respective *H*_e _and life forms as dependent variables. In the second model, the phylogenetic relationships of species were incorporated as a correlating matrix, obtained from the phylogenetic tree above, into the GLM by using the generalized estimating equation (GEE) procedure. Such a procedure is generally used to fit the parameters of a GLM when the observations are correlated or non-independent. In our particular case, the common ancestry of species is a source of non-independence, which was taken into account with the inclusion of the above-mentioned matrix. The third model was similar to the second one, but on it, it was assumed that conifers and angiosperm trees were different "life-forms". The GEE procedure used in these last two models was the one implemented in the APE package [55].

### Estimation of recombination rates based on nuclear gene DNA sequences

Original DNA sequences from nuclear genes of non-domesticated species were downloaded from GenBank or obtained directly from the authors (totalling ~2.5 Mbp distributed in 43 genes from eight species), and edited and aligned with Lasergen SeqMan vs. 7 (DNASTAR, Madison, USA). Domesticated taxa were deliberately excluded because most studies in these species focused on genes related to domestication, which typically show low levels of polymorphism and have followed artificial selection. Only those sequences spanning at least 800 base pairs (bp), having a minimum of 10 segregating sites, and sampled for more than 20 chromosomes were taken into account. Similarly, only DNA sequences from regions with low genetic differentiation or from single populations were used for those species with known population structure. For example, only sequences from Sweden were considered for *Populus tremula *or from Balsas for *Zea mays *ssp. *parviglumis*.

The aligned contigs were then used to estimate different diversity and recombination parameters such as the average number of nucleotide differences (*θ*_*π*_), the minimum number of recombination events (*R*m) [56], the population-scaled recombination rate (*ρ*), and the recombination to mutation ratio (*θ*/*ρ*). The first two statistics were computed using DnaSP vs. 4.2 [57], while two different estimates of *ρ *and *θ*/*ρ *were calculated with the composite-likelihood method of Hudson [58] implemented in LDhat [59], and with the summary statistics method available in the *rhothetapost *software [60]. Contrary to the first approach, the summary statistics method allows the co-estimation of mutation and recombination rates, and the computation of 95% confidence intervals based on the posterior distribution of these parameters. These analyses were made exclusively on parsimoniously informative sites, and disregarding indels and polymorphisms with more than two states. Raw estimates of nucleotide diversity and recombination parameters were finally taken from the original references for other species such as *Quercus crispula *[61] and *Hordeum spontaneum *[3] and included in the comparisons.

## Abbreviations

cM: centimorgan; *f*: ratio of gene conversion to cross-over; *H*_e_: mean expected heterozygosity; Mb: megabase; PIC: phylogenetically independent contrast; SSR: simple sequence repeats.

## Authors' contributions

JPJ-C conceived the study, collected the data and wrote the manuscript, MV conceived the study, analysed the data, and edited the manuscript, SCGM conceived and coordinated the study, analysed the data, and edited the manuscript. All authors read and approved the final version of the manuscript.

## Supplementary Material

Additional file 1 Number of chromosomes and estimates of genetic map length, physical genome size, genome-wide rate of recombination and mean expected heterozygosity at SSR's (*H*_e_) for 81 higher plant species classified according to their type of life-form. The rates of recombination were corrected following Hall & Willis (2005). Click here for file

Additional file 2 Comparison of estimates of nucleotide diversity and recombination rates across different types of wild plant life-forms based on nuclear gene DNA sequences from population studies. Click here for file
