# Supplementary material for: The contribution of recombination to heterozygosity differs among plant evolutionary lineages and life-forms
Source: BMC Evol Biol. 2010 Jan 25;10:22. doi: 10.1186/1471-2148-10-22 (PMC2826329; doi:10.1186/1471-2148-10-22)
Supplement: Additional file 1 — Number of chromosomes and estimates of genetic map length, physical genome size, genome-wide rate of recombination and mean expected heterozygosity at SSR's (He) for 81 higher plant species classified according to their type of life-form. The rates of recombination were corrected following Hall & Willis (2005). [file 1471-2148-10-22-S1.DOC]

**Additional file 1:** Number of chromosomes and estimates of genetic map length, physical genome size, genome-wide rate of recombination and mean expected heterozygosity at SSR’s (*H*_e_) for 81 higher plant species classified according to their type of life-form. The rates of recombination were corrected following Hall & Willis (2005).

| Life-form /  Species | Familly | No. Chrom. | Map length (cM) | Genome size (Mb) | Recomb. rate (cM/Mb) | Corrected recomb. rate (cM/Mb) | *H*_e_ | References^a^ |
| --- | --- | --- | --- | --- | --- | --- | --- | --- |
| Herbs |  |  |  |  |  |  |  |  |
| *Spinacia oleracea* | Amaranthaceae | 12 | 585.0 | 1007.3 | 0.581 | 0.593 | 0.680 | 69, 70 |
| *Daucus carota* | Apiaceae | 18 | 534.4 | 978.0 | 0.546 | 0.567 | 0.236 | 16, 140 |
| *Catharanthus roseus* | Apocynaceae | 16 | 1131.9 | 2376.5 | 0.476 | 0.488 | 0.560 | 55, 122 |
| *Asparagus officinalis* | Asparagaceae | 22 | 721.4 | 1320.3 | 0.546 | 0.553 | 0.530 | 1, 125 |
| *Helianthus agnus* | Asteraceae | 34 | 828.0 | 2376.5 | 0.348 | 0.351 | 0.670 | 21, 54 |
| *Helianthus petiolaris* | Asteraceae | 34 | 1592.0 | 3325.2 | 0.479 | 0.487 | 0.660 | 21, 54 |
| *Cynara cardunculus* | Asteraceae | 34 | 1330.5 | 1075.8 | 1.237 | 1.232 | 0.499 | 3, 78 |
| *Cichorium intybus var. foliosum* | Asteraceae | 18 | 609.6 | 978.0 | 0.623 | 0.638 | 0.237 | 50, 135 |
| *Brassica oleracea* | Brassicaceae | 18 | 1606.0 | 762.8 | 2.105 | 2.11 | 0.775 | 20, 33 |
| *Arabidopsis lyrata* | Brassicaceae | 16 | 515.0 | 230.0 | 2.239 | 2.241 | 0.310 | 34, 75 |
| *Arabidopsis thaliana* | Brassicaceae | 10 | 520.0 | 123.5 | 4.211 | 4.211 | 0.060 | 58, 136 |
| *Ananas comosus* | Bromeliaceae | 50 | 4146.0 | 537.9 | 7.708 | 7.624 | 0.509 | 23, 71 |
| *Humulus lupulus* | Cannabaceae | 20 | 661.9 | 2836.2 | 0.233 | 0.245 | 0.672 | 26, 65 |
| *Cucumis melo* | Cucurbitaceae | 24 | 1654.0 | 929.1 | 1.780 | 1.781 | 0.532 | 102, 123 |
| *Cucurbita pepo* | Cucurbitaceae | 40 | 1936.0 | 537.9 | 3.599 | 3.601 | 0.620 | 51, 52 |
| *Vicia faba* | Fabaceae | 24 | 1559.0 | 13036.7 | 0.119 | 0.12 | 0.480 | 66, 111 |
| *Trifolium pratense* | Fabaceae | 14 | 444.2 | 635.7 | 0.699 | 0.702 | 0.880 | 60, 72 |
| *Vigna angulares* | Fabaceae | 22 | 832.1 | 518.3 | 1.605 | 1.597 | 0.170 | 57, 141 |
| *Vigna radiata* | Fabaceae | 22 | 737.9 | 1271.4 | 0.580 | 0.584 | 0.313 | 62, 124 |
| *Antirrhinum molle* | Plantaginaceae | 16 | 613.0 | 1564.8 | 0.392 | 0.401 | 0.610 | 119, 147 |
| *Antirrhinum majus* | Plantaginaceae | 16 | 613.0 | 1564.8 | 0.392 | 0.401 | 0.450 | 119, 147 |
| *Zizania palustres* | Poaceae | 30 | 1805.0 | 2132.1 | 0.847 | 0.846 | 0.558 | 68, 108 |
| *Oryza sativa* | Poaceae | 24 | 1575.0 | 489.0 | 3.221 | 3.221 | 0.700 | 49, 77 |
| *Lolium perenne* | Poaceae | 14 | 811.0 | 2034.2 | 0.398 | 0.396 | 0.560 | 46, 126 |
| *Zea mays* | Poaceae | 20 | 1784.7 | 2669.9 | 0.668 | 0.67 | 0.890 | 35, 126 |
| *Zea luxurians* | Poaceae | 20 | 1397.0 | 4479.2 | 0.312 | 0.308 | 0.730 | 48, 84 |
| *Zea diploperennis* | Poaceae | 20 | 1247.0 | 2591.7 | 0.481 | 0.479 | 0.650 | 48, 143 |
| *Festuca pratensis* | Poaceae | 14 | 658.8 | 8704.2 | 0.076 | 0.074 | 0.370 | 4, 48 |
| *Triticum monococcum* | Poaceae | 14 | 1067.0 | 5868.0 | 0.182 | 0.183 | 0.423 | 44, 88 |
| *Hordeum vulgare* | Poaceae | 14 | 925.6 | 5418.1 | 0.171 | 0.171 | 0.450 | 83, 131 |
| *Hordeum spontaneum* | Poaceae | 14 | 445.0 | 5379.0 | 0.083 | 0.083 | 0.520 | 61, 137 |
| *Fagopyrum esculentum* | Polygonaceae | 16 | 508.3 | 1359.4 | 0.374 | 0.377 | 0.819 | 64, 144 |
| *Fagopyrum homotropicum* | Polygonaceae | 16 | 548.9 | 1095.4 | 0.501 | 0.512 | 0.714 | 73, 145 |
| *Lycopersicum peruvianum* | Solanaceae | 24 | 1073.0 | 1124.7 | 0.954 | 0.96 | 0.568 | 5, 138 |
| *Lycopersicum esculetum* | Solanaceae | 24 | 1469.0 | 950.0 | 1.546 | 1.549 | 0.034 | 5, 129 |
| Mean | --- | 21.26 | 1099.61 | 2350.9 | 1.152 | 1.153 | 0.527 |  |
| Standard error | --- | 0.25 | 20.14 | 75.68 | 0.044 | 0.043 | 0.006 |  |

**Table S1.** Continued …

| Life-form /  Species | Familly | No. Chrom. | Map length (cM) | Genome size (Mb) | Recomb. rate (cM/Mb) | Corrected recomb. rate (cM/Mb) | *H*_e_ | References^a^ |
| --- | --- | --- | --- | --- | --- | --- | --- | --- |
| Shrubs |  |  |  |  |  |  |  |  |
| *Manihot esculenta* | Euphorbiaceae | 36 | 1236.7 | 1633.3 | 0.757 | 0.761 | 0.460 | 94, 96 |
| *Gossypium raimondii* | Malvaceae | 26 | 1493.3 | 1323.0 | 1.129 | 1.135 | 0.655 | 31, 112 |
| *Rosa wichuraiana* | Rosaceae | 14 | 438.0 | 567.2 | 0.772 | 0.78 | 0.276 | 97, 113 |
| *Rubus idaeus idaeus* | Rosaceae | 14 | 505.0 | 332.5 | 1.519 | 1.525 | 0.620 | 115, 116 |
| *Camellia sinensis* | Theaceae | 30 | 1349.7 | 3814.2 | 0.354 | 0.354 | 0.499 | 56, 67 |
| *Passiflora edulis f. flaviocarpa* | Passifloraceae | 18 | 790.2 | 1418.1 | 0.557 | 2.085 | 0.440 | 81, 95 |
| *Vitis rupestris* | Vitaceae | 38 | 756.0 | 537.9 | 1.405 | 0.267 | 0.720 | 8, 43 |
| *Vitis vinifera* | Vitaceae | 38 | 1245.0 | 475.0 | 2.621 | 0.822 | 0.580 | 8, 133 |
| Mean | --- | 26.75 | 976.74 | 1262.65 | 1.139 | 1.144 | 0.531 |  |
| Standard error | --- | 1.29 | 50.48 | 143.07 | 0.090 | 0.090 | 0.017 |  |
|  |  |  |  |  |  |  |  |  |
|  |  |  |  |  |  |  |  |  |
| Trees (Angiosperms) |  |  |  |  |  |  |  |  |
| *Anacardium occidentale* | Anacardiaceae | 42 | 1050.7 | 492.0 | 2.136 | 1.769 | 0.420 | 25, 37 |
| *Asimina triloba* | Annonaceae | 18 | 206.0 | 782.4 | 0.264 | 1.74 | 0.358 | 104, 142 |
| *Cocos nucifera* | Arecaceae | 32 | 2226.0 | 2744.0 | 0.811 | 1.138 | 0.547 | 59, 110 |
| *Elaeis guineensis* | Arecaceae | 32 | 1743.0 | 978.0 | 1.782 | 8.539 | 0.680 | 13, 14 |
| *Corylus avellana* | Betulaceae | 22 | 812.0 | 469.4 | 1.729 | 0.129 | 0.780 | 15, 89 |
| *Betula pendula* | Betulaceae | 28 | 1561.0 | 1369.2 | 1.140 | 1.008 | 0.670 | 76, 100 |
| *Carica papaya* | Caricaceae | 18 | 3294.2 | 372.0 | 8.855 | 1.526 | 0.570 | 82, 93 |
| *Hevea spp.* | Euphorbiaceae | 36 | 2144.0 | 2102.7 | 1.019 | 1.77 | 0.741 | 80, 114 |
| *Acacia mangium* | Fabaceae | 26 | 966.0 | 635.7 | 1.519 | 1.021 | 0.510 | 22, 91 |
| *Castanea sativa* | Fagaceae | 24 | 865.0 | 792.2 | 1.092 | 2.086 | 0.724 | 24, 86 |
| *Fagus sylvatica* | Fagaceae | 24 | 971.0 | 547.7 | 1.773 | 1.618 | 0.691 | 117, 98 |
| *Quercus robur* | Fagaceae | 24 | 921.7 | 909.5 | 1.013 | 2.431 | 0.898 | 11, 39 |
| *Carya illinoinensis* | Juglandaceae | 16 | 1698.0 | 811.7 | 2.092 | 3.099 | 0.654 | 12, 53 |
| *Morus spp.* | Moraceae | 28 | 1351.7 | 831.3 | 1.626 | 2.001 | 0.430 | 139, 146 |
| *Eucalyptus globulus* | Myrtaceae | 22 | 1375.0 | 567.2 | 2.424 | 1.289 | 0.850 | 17, 130 |
| *Eucalyptus grandis* | Myrtaceae | 22 | 1814.5 | 567.2 | 3.199 | 0.108 | 0.890 | 17, 18 |
| *Eucalyptus urophylla* | Myrtaceae | 22 | 1133.4 | 567.2 | 1.998 | 0.095 | 0.830 | 17, 19 |
| *Olea europea* | Oleaceae | 46 | 2445.0 | 1907.1 | 1.282 | 0.061 | 0.550 | 40, 107 |
| *Macadamia integrifolia* | Proteaceae | 28 | 1100.0 | 811.7 | 1.355 | 2.367 | 0.526 | 99, 118 |
| *Prunus davidiana* | Rosaceae | 16 | 536.0 | 273.8 | 1.957 | 1.31 | 0.450 | 41, 47 |
| *Prunus armeniaca* | Rosaceae | 16 | 562.0 | 293.4 | 1.916 | 1.123 | 0.650 | 42, 132 |
| *Coffea liberica* | Rubiaceae | 32 | 1502.5 | 635.7 | 2.364 | 2.114 | 0.350 | 38, 90 |
| *Coffea canephora* | Rubiaceae | 22 | 1041.0 | 800.0 | 1.301 | 5.047 | 0.290 | 38, 79 |
| *Poncirus trifoliata* | Rutaceae | 18 | 425.7 | 381.4 | 1.116 | 7.741 | 0.625 | 10, 32 |
| *Citrus sinensis* | Rutaceae | 18 | 775.8 | 367.0 | 2.114 | 5.236 | 0.519 | 32, 92 |
| *Populus deltoides* | Salicaceae | 38 | 2520.0 | 500.0 | 5.040 | 4.579 | 0.281 | 29, 105 |

**Table S1.** Continued …

| Life-form /  Species | Familly | No. Chrom. | Map length (cM) | Genome size (Mb) | Recomb. rate (cM/Mb) | Corrected recomb. rate (cM/Mb) | *H*_e_ | References^a^ |
| --- | --- | --- | --- | --- | --- | --- | --- | --- |
| *Populus nigra* | Salicaceae | 38 | 3869.0 | 500.0 | 7.738 | 3.761 | 0.440 | 28, 105 |
| *Populus trichocarpa* | Salicaceae | 38 | 2616.0 | 500.0 | 5.232 | 2.107 | 0.302 | 27, 105 |
| *Salix viminalis* | Salicaceae | 38 | 1844.0 | 400.9 | 4.598 | 0.567 | 0.615 | 9, 134 |
| *Populus tremula* | Salicaceae | 38 | 1875.0 | 500.0 | 3.750 | 1.408 | 0.448 | 36, 87 |
| *Theobroma cacao* | Sterculiaceae | 20 | 885.4 | 420.5 | 2.105 | 2.623 | 0.372 | 109, 120 |
| Mean (angiosperm trees) | --- | 27.16 | 1488.08 | 768.75 | 2.463 | 2.449 | 0.569 |  |
| Standard error (angiosperm trees) | --- | 0.28 | 27.28 | 17.93 | 0.063 | 0.062 | 0.006 |  |
|  |  |  |  |  |  |  |  |  |
| Trees (conifers) |  |  |  |  |  |  |  |  |
| *Cryptomeria japonica* | Cupressaceae | 22 | 1372.2 | 10758.0 | 0.128 | 1.087 | 0.770 | 127, 128 |
| *Picea abies* | Pinaceae | 24 | 1856.0 | 18190.8 | 0.102 | 0.065 | 0.790 | 2, 103 |
| *Picea glauca* | Pinaceae | 24 | 1837.5 | 19755.6 | 0.093 | 0.091 | 0.660 | 101, 106 |
| *Pinus pinaster* | Pinaceae | 24 | 1441.0 | 23814.3 | 0.061 | 0.323 | 0.752 | 30, 85 |
| *Pinus taeda* | Pinaceae | 24 | 1339.5 | 21516.0 | 0.062 | 1.357 | 0.513 | 45, 121 |
| *Pseudotsuga menziesii* | Pinaceae | 26 | 1664.0 | 18630.9 | 0.089 | 1.961 | 0.673 | 6, 74 |
| *Larix kaempferi* | Pinaceae | 24 | 2997.0 | 9291.0 | 0.323 | 1.92 | 0.756 | 7, 63 |
| Mean (conifers) | --- | 24 | 1786.74 | 17422.37 | 0.123 | 0.125 | 0.702 |  |
| Standard error (conifers) | --- | 0.16 | 82.04 | 772.93 | 0.013 | 0.012 | 0.014 |  |
|  |  |  |  |  |  |  |  |  |
| Total Mean (Trees) | --- | 26.58 | 1543.10 | 3836.52 | 2.032 | 2.021 | 0.594 |  |
| Standard error (Trees) | --- | 0.21 | 21.17 | 181.95 | 0.053 | 0.052 | 0.005 |  |

^a^ **References:**

1. Aceto S, et al. (2003) Isolation and characterization of microsatellite loci from *Asparagus acutifolius* (Liliaceae). Mol Ecol Notes 3:242-243.

2. Acheré V, et al. (2004) A full saturated linkage map of *Picea abies* including AFLP, SSR, EST, 5S rDNA and morphological markers. Theor Appl Genet 108:1602-1613.

3. Acquadro A, Portis E, Lee D, Donini P, Lanteri S (2005) Development and characterization of microsatellite markers in *Cynara cardunculus* L. Genome 48:217-225.

4. Alm V et al. (2003) A linkage map of meadow fescue (*Festuca pratensis* Huds.) and comparative mapping with other Poaceae species. Theor Appl Genet 108:25-40.

5. Alvarez AE, van de Wiel CCM, Smulder JMJ, Vosman B (2001) Use of microsatellites to evaluate genetic diversity and species relationships in the genus *Lycopersicon*. Theor Appl Genet 103:1283-1292.

6. Amarasinghe V, Carlson JE (2002) The development of microsatellite DNA markers for genetic analysis in Douglas-fir. Can J For Res 32:1904-1915.

7. Arcade A et al. (2000) Application of AFLP, RAPD and ISSR markers to genetic mapping of European and Japanese larch. Theor Appl Genet 100:299-307.

8. Arrigo N, Arnold C (2007) Naturalised *Vitis* rootstocks in Europe and consequences to native wild grapevine. PLoS ONE 2:e521.

9. Barker JHA, Pahlich A, Trybush S, Edwards KJ, Karp A (2003) Microsatellite markers for diverse *Salix* species. Mol Ecol Notes 3:4-6.

10. Barkley NA, Roose ML, Krueger RR, Federici CT (2006) Assessing genetic diversity and population structure in a citrus germplasm collection utilizing simple sequence repeat markers (SSRs). Theor Appl Genet 112:1519-1531.

11. Barreneche T, et al. (1998) A genetic linkage map of *Quercus robus* L. (pedunculate oak) based on RAPD, SCAR, microsatellite, minisatellite, isozyme, and 5S rDNA markers. Theor Appl Genet 97:1090-1103. 12. Beenadagri SR, Dove SK, Wood BW, Corner PJ (2005) A first linkage map of pecan cultivars based on RAPD and AFLP markers. Theor Appl Genet 110:1127-1137.

13. Billotte N, et al. (2001) Development, characterization and across-taxa utility of oil palm (*Elaeis guineensis* Jacq.) microsatellite markers. Genome 44:413-425.

14. Billotte N, et al. (2005) Microsatellite-based high density linkage map in oil palm (*Elaeis guineensis* Jacq.) Theor Appl Genet 110:754-765.

15. Boccacci P, Akkak A, Bassil V, Mehlenbacher A, Botta R (2005) Characterization and evaluation of microsatellite loci in European hazelnut (*Corylus avellana* L.) and their transferability to other *Corylus* species. Mol Ecol Notes 5:934-937.

16. Briard M, Le Clerc V, Mausset AE, Veeret A (2000) A comparative study on the use of ISSR, microsatellite and RAPD markers for varietal identification of carrot genotypes. Acta Hort 546:377-385.

17. Brondani, RPV, Brondani C, Tarchini R, Grattapaglia D (1998) Development, characterization and mapping of microsatellite markers in *Eucalyptus grandis* and *E. urophylla*. Theor Appl Genet 97:816-827.

18. Brondani, RPV, Williams ER, Brondani C, Grattapaglia D (2006) A microsatellite-based consensus linkage map for species of *Eucalyptus* and a novel set of 230 microsatellite markers for the genus. BMC Plant Biol 6:20.

20. Burges B, et al. (2006) Identification and characterization of simple sequence repeat (SSR) markers derived *in silico* from *Brassica oleracea* genome shotgun sequences. Mol Ecol Notes 6:1191-1194.

21. Burke JM, et al. (2004) Comparative mapping and rapid karyotypic evolution in the genus *Helianthus*. Genetics 167:449-457.

22. Butcher PA, Moran GF (2000) Genetic linkage mapping in *Acacia magnium*. 2. Development of an integrated map from two outbreed pedigrees using RFLP and microsatellite loci. Theor Appl Genet 101:594-605.

23. Carlier JD, Reis A, Duval MF, Coppens D’eeckenbrugge G, Leitão JM (2004) Genetic maps of RAPD, AFLp and ISSR markers in *Ananas bracteatus* and *A. comosus* using the pseudo-testcross strategy. Plant Breed 123:186-192.

24. Casasoli M, Mattioni C, Cherubini M, Villani F (2001) A genetic linkage map of European chesnut (*Castanea sativa* Mill.) based on RAPD, ISSR, and isozyme markers. Theor Appl Genet 102:1190-1199.

25. Cavalcanti JJV, Wilkinson MJ (2007) The first genetic maps of cashew (*Anacardium occidentale* L.). Euphytica 157:131-143.

26. Cerenak A, Satovic Z, Javornik B (2006) Genetic mapping of hop (*Humulus lupulus* L.) applied to the detection of QTLs for alpha-acid content. Genome 49:485-494.

27. Cervera MT, et al. (2001) Dense genetic linkage maps of three populus species (*Populus deltoides*, *P. nigra*, and *P. trichocarpa*) based on AFLP and microsatellite markers. Genetics 158:787-809.

30. Chagné D (2002) A high density genetic map f maritime pine based on AFLPs. Ann For Sci 59:627-636.

31. Changbiao W, Wangzhen G, Caiping C, Tianzhen Z (2006) Characterization, development and exploitation of EST-derived microsatellites in *Gossypium raimondii* Ulbrich. Chinese Sci Bull 57:557-561.

32. Chen C, et al. (2008) EST-SSR genetic maps for *Citrus sinensis* and *Poncirus trifoliata*. Tree Genet Genome 4:1-10.

33. Cheung WY, Champagne G, Hubert N, Landry BS (1997) Comparison of the genetic maps of *Brassica napus* and *Brassica oleracea*. Theor Appl Genet 94:569-582.

34. Clauss MJ, Cobban H, Mitchell-Olds T (2002) Cross-species microsatellite markers for elucidating population genetic structure in *Arabidopsis* and *Arabis* (Brassicacea). Mol Ecol 11:591-601.

35. Coe E, et al. (2002) Access to the Maize genome: an integrated physical and genetic map. Plant Physiol 128:9-12.

36. Cole CT (2006) Allelic and population variation of microsatellite loci in aspen (*Populus tremuloides*). New Phytol 167:155-164.

37. Croxford AE, Robson M, Wilkinson MJ (2006) Characterization and PCR multiplexing of polymorphic microsatellite loci in cashew (*Anacardium occidentale* L.) and their cross-species utilization. Mol Ecol Notes6: 249-251.

38. Cubry P, et al. (2008) Diversity in coffee assessed with SSR markers: structure of the genus *Coffea* and perspectives for breeding. Genome 51:50-63.

39. Degen B, Streiff R, Ziegenhagen B (1999) Comparative study of genetic variation and differentiation of two pedunculate oak (*Quercus robur*) stands using microsatellite and allozyme loci. Heredity 83:597-603.

40. de la Rosa R, et al. (2003) A first genetic map of olive (*Olea europea* L.) cultivars using RAPD, AFLP, RFLP and SSR markers. Theor Appl Genet 106:1273-1282.

41. Dirlewanger E, et al. (2004) comparative mapping and marker-assisted selection in Rosaceae fruit crops. Proc Natl Acad Sci USA 101:9891-9896.

42. Dondini L, et al. (2007) Develpoment of a new SSR-based linkage map in apricot and analysis of synteny with exiting *Prunus* maps. Tree Genet Genome 3:239-249.

43. Ducleff M, et al. (2004) A genetic linkage map of grape, utilizing *Vitis rupestris* and *Vitis arizonica*. Theor Appl Genet 109:1178-1187.

44. Dubocovsky J, et al. (1996) Genetic map of diploid wheat, *Triticum monococcum* L., and its comparison with maps of *Hordeum vulgare* L. Genetics 143:983-99.

45. Elsik CG; Minihan VT, Hall SE, Scarpa AM, Williams CG (2000) Low-copy microsatellite maerkers for *Pinus taeda* L. Genome 43:550-555.

46. Faville MJ, et al. (2004) Functionally associated molecular genetic map construction in perennial rye grass (*Lolium perenne* L.). Theor Appl Genet 110:12-32.

47. Foulongne M, Minier J, Pascal T, Kervella J (2003) Genetic relationships between peach (*Prunus persica* L.) and closely related wild species using SSR markers. Acta Hort 663:629-634.

48. Fukunaga K, et al. (2005) Genetic diversity and population structure of teosinte. Genetics 169:2241-2254.

49. Garris, AJ et al. (2005) Genetic structure and diversity in *Oryza sativa* L. Genetics 169:1631-1638.

50. Gemeinholzer B, Bachmann K (2005) Examining morphological and molecular diagnostic character states of *Cichorium intybus* L. (Asteraceae) and *C. spinosum* L. Plant Syst Evol 253:105-123.

51. Gong L et al. (2008) Microsatellites for the genus *Cucurbita* and an SSR –based genetic linkage map of *Cucurbita pepo* L. Theor Appl Genet 117:37-48.

53. Grauke et al. Unpublished data.

54. Gross BL, Schwarzbach AE, Rieseberg LE (2003) Origin(s) of the diploid hybrid species *Helianthus deserticola* (Asteraceae). Am J Bot 90:1708-1719.

55. Gupta S et al., (2007) Construction of genetic linkage map of the medicinal and ornamental plant *Catharanthus roseus*. J Genet 86:259-268.

56. Hackett GA et al., (2000) Construction of a genetic linkage map for *Camellia sinensis* (tea). Heredity 85:346-355.

57. Han VA et al., (2005) A genetic linkage map for azuki bean [*Vigna angularis* (Wild.) Ohwi & Ohashi]. Theor Appl Genet 111:1278-1287.

58. Hauge BM et al., (1993) An integrated genetic/RFLP map of the *Arabidopsis thaliana* genome. Plant J 3:745-754.

59. Herrán A et al., (2000) Linkage mapping and QTL analysis of coconut (*Cocos nucifera* L.). Theor Appl Genet 101:292-300.

60. Hermann D et al. (2006) QTL of seed yield components in red clover (*Trifolium pratense* L.). Theor Appl Genet 112:536-545.

61. Huang Q et al. (2002) Mosaic microecological differential stress causes adaptive microsatellite divergence in wild barley, *Hordeum spontaneum*,. At Neve Yaar, Israel. Genome 45:1216-1229.

62. Humphry M et al., (2002) Development of a mungbean (*Vigna radiata*) RFLP linkage map and its comparison with Lablab (*Lablab purpureus*) reveals a high level of colinearity between the two genomes. Theor Appl Genet 105:160-166.

63. Isoda K, Watanabe A (2006) Isolation and characterization pf microsatellite loci from *Larix kaempferi*. Mol Ecol Notes 6:664-666.

64. Iwata H, Imon K, Tsumura Y, Ohsawa R (2005) Genetic diversity among Japanese indigenous common buckwheat (*Fagopyrum esculentum*) cultivars as determined from amplified fragment length polymorphisms and simple sequence repeat markers and quantitative agronomic traits. Genome 48:367-377.

65. Jakše J, Javornik B (2001) High throughput isolation of microsatellites in hop (*Humulus lupulus* L.). Plant Mol Biol Rep. 19:217-226.

66. Kalia P, Sood S (2004) Genetic variation and association analyses for pod yield and other agronomic and quality characters in an Indian and Himalayan collection of broad bean (*Vicia faba* L.). SABRAO J Breed Genet 36:55-61.

67. Kaudun S, Matsumoto S (2002) Heterologous nuclear and chloroplast microsatellite amplification and variation in tea, *Camellia sinensis*. Genome 45:1041-1048.

68. Kennard W et al., (1999) A comparative map of wild rice (*Zizania palustris* L. 2n=2x=30). Theor Appl Genet 99:793-799.

69. Khattak JZK, Torp AM, Andersen SB (2006) A genetic linkage map of *Spinacia oleracea* and localization of sex determination locus. Euphytica 348:311-318.

70. Khattak JZK, Christianses JL, Torp AM, Andersen SB (2007) Genic microsatellite markers for discrimination of spinach cultivars. Plant Breed 126:454-456.

71. Kinsuat MJ, Kumar SV (2007) polymorphic microsatellite and cryptic simple sequence repeat sequence markers in pineapples (*Ananas comosus* var. *comosus*). Mol Ecol Notes 7:1032-1035.

72. Kölliker r, et al. (2006) Development and characterisation of simple sequence repeat (SSR) markers for white clover (*Trifolium repens* L.). Theor Appl Genet 102:416-424.

73. Konishi T, et al. (2006) Development and characterisation of microsatellite markers for common buckwheat. Breed Sci 36:277-285.

74. Krutovsky KV, et al. (2004) Comparative mapping in the Pinaceae. Genetics 168:447-461.

75. Kuittinen H, et al. (2004) Comparing the linkage maps of the close relatives *Arabidopsis lyrata* and *A. thaliana*. Genetics 168:1575-1584.

76. Kulju KKM, Pekkinen M, Varvio S (2004) Twenty-three microsatellite primer pairs for *Betula pendula* (Betulaceae). Mol Ecol Notes 4:471-473.

77. Kurata N, et al. (1994) A 300 kilobase intervale genetic map of rice including 883 expressed sequences. Nat Genet 8:365-372.

78. Lanteri S, et al. (2006) A first linkage map of globe artichoke (*Cynara cardunculus* var *scolymus* L.) based on AFLP, S-SAP, M-AFLP, and microsatellite markers. Theor Appl Genet 118:1532-1542.

79. Lashermes P, et al. (2001) Genetic linkage map of *Coffea canephora*: effect of segregation distortion and analysis of recombination rate in male and female meioses. Genome 44:589-596.

80. Lespinasse D, et al. (2000) A saturated genetic linkage map of rubber tree (*Hevea* spp.) based on RFLP, AFLP, microsatellite and isozyme markers. Theor Appl Genet 100:127-138.

81. Lopes R, et al. (2006) Linkage and mapping of resistance genes to *Xanthomonas axonopodis* pv. *passiflorae* in yellow passion fruit. Genome 49:17-29.

82. Ma H, et al. (2004) High-desinty linkage mapping revealed suppression of recombination at the sex determination locus in papaya. Genetics 166:419-436.

83. Mano Y, Kawasaki S, Takaiwa F, Komatsuda T (2001) construction of a genetic map of barley (*Hordeum vulgare* L.) cross ‘Asumamugi’ x ‘Kanot nakate gold’ using a simple and efficient amplified fragment-length polymorphism system. Genome 44:284-292.

84. Mano Y, Omori F, Kindiger B, Takahashi H (2008) A linkage map of maize x teosinte *Zea luxurians* and identification of QTL controlling root aerenchyma formation. Mol Breed 21:327-337.

85. Mariette S, et al. (2001) Microsatellite markers for *Pinus pinaster* Ait. Ann For Sci 58:203-206.

86. Marinoni D, et al. (2003) Development and characterization of microsatellite markers in *Castanea sativa* (Mill.). Mol Breed 11:127-136.

87. Markussen T, Pakull B, Fladung M (2007) Positioning of sex-correlated markers for Populus in a AFLP- SSR-marker based genetic map of *Populus tremula* x *tremuloides*. Silvae Genet 56:180-184.

88. McLauchlan A, et al. (2001) Development of robust PCR-based DNA markers for each homeo-allele of granule-bond starch synthase and their application in wheat breeding programs. Aus J Agric Res 52:1409-1416.

89. Mehlenbacher SA, et al. (2006) A genetic linkage map of hazelnut (*Corylus avellana* L.) based on RAPD and SSR markers. Genome 49:122-133.

90. N'Diaye A, Noirot M, Hamon S, Poncet V (2007) Genetic basis of species differentiation between *Coffea liberica* Hiern and *C. canephora* Pierre: analysis of an interspecific cross. Genet Res Crop Evol 54:1011-1021.

91. Ng CH, et al. (2005) Isolation of 15 polymorphic microsatellite loci in *Acacia* hybrid (*Acacia mangium* x *A. auricufolius*). Mol Ecol Notes 5:572-575.

92. Novelli VM, Cristofani M, Souza AA, Machado MA (2006) Development and characterization of polymorphic microsatellite markers for the sweet orange (Citrus sinensis L. Osbeck). Genet Mol Biol 29:90-96.

93. Ocampo-Perez J, et al. (2006) Microsatellite markers in *Carica papaya* L.: isolation, characterization and transferability to *Vasconcellea* species. Mol Ecol Notes 6:212-217.

94. Okogbenin E, Marin J, Fregene M (2006) An SSR-based molecular genetic map of cassava. Euphytica 147:433-440.

95. Oliveira EJ, et al. (2005) Development and characterization of microsatellite markers from the yellow passion fruit (Passiflora edulis f. flaviocarpa). Mol Ecol Notes 5:331-333.

96. Olsen KM, Schaal BA (2001) Microsatellite variation in cassiva (*Manihot esculenta*,Euphorbiaceae) and its wild relatives: further evidence for a southern Amazonian origin of domestication. Am J Bot 88:131-142.

97. Oyant LH-S, et al. (2008) Genetic linkage maps of rose constructed with new microsatellite markers and locating QTL controlling flower traits. Tree Genet Genome 4:11-23.

98. Pastorelli R, et al. (2003) Characterization of microsatellite markers in *Fagus sylvatica* L. and *Fagus orientalis* Lipsky. Mol Ecol Notes 3:76-79.

99. Peace CP, Vithanage V, Turnbull CGN, Caroll BJ (2003) A genetic map of macadamia based on randomly amplified DNA fingerprinting RAF methods. Euphytica 134:17-26.

100. Pekkinen P, et al. (2005) Linkage map of birch, *Betula pendula* Roth, based on microsatellites and amplified fragment length polymorphisms. Genome 48: 619-625.

101. Pelgas B, et al. (2006) Comparative genome mapping among *Picea glauca*, *P. mariana* x *P. rubens* and *P. abies*, and correspondence with other Pinaceae. Theor Appl Genet 113:1371-1393.

102. Périn C, et al. (2002) A reference map of *Cucumis melo* based on two recombinant inbred line populations. Theor Appl Genet 104:1017-1034.

103. Pfeiffer A, Olivieri AM, Morgante M (1997) Identification and characterization of microsatellites in Norway spruce (*Picea abies* K.) Genome 40:411-419.

104. Pomper KW, et al. (2003) Assessment of genetic diversity of pawpaw (*Asimina triloba*) cultivars intersimple sequence repeat markers. J Amer Soc Hort Sci 128:521-525.

105. Rahman MH, Rajora OP (2002) Microsatellite DNA fingerprinting, differentiation, and genetic relationships of clones, cultivars, and varieties of six poplar species from three sections of the genus *Populus*. Genome 45:1083-1094.

106. Rajora OP, Rahman RH, Dayanandan S, Mosseler A (2001) Isolation, characterization, inheritance, and linkage of microsatellite DNA markers in white spruce (*Picea glauca*) and their usefulness in other spruce species. Mol General Genet 264:871-882.

107. Rallo R, Dorado G, Martin A (2000) Development of simple sequence repeats (SSRs) in olive trees (*Olea europaea* L.) Theor Appl Genet 101:984-989.

108. Richards CM, Reilley A, Touchell D, Antolin MF, Walters C (2004) Microsatellite primers for Texas wild rice (*Zizania*), and a preliminary test of impact of cryogenetic storage on allele frequency at these loci. Cons Genet 5:853-859.

109. Risterucci AM, et al. (2000) A high-density linkage map of *Theobroma cacao* L. Theor Appl Genet 101:948-955.

110. Rivera R, et al. (1999) Isolation and characterization of polymorphic microsatellites in *Cocos nucifera* L. Genome 42:668-675.

111. Román B, et al. (2002) Mapping of quantitative trait loci controlling broomrape (*Orobanche crenata* Forsk.) resistance in faba bean (*Vicia faba* L.). Genome 45:1057-1063.

112. Rong J, et al. (2004) A 3347-locus genetic recombination map of sequence-tagged sites reveals features of genome organization, transmission and evolution of cotton (*Gossypium*). Genetics 166: 389-417.

113. Rusanov K, et al. (2005) Microsatellite analysis of *Rosa damacena* Mill. accessions reveals genetic similarity between genotypes used for rose oil production and old Damasak rose varieties. Theor Appl Genet 11:804-809.

114. Saha T, Bindu Roy T, Nazeer MA (2005) Microsatellite variability an its use in the characterization of cultivated clones of Hevea brasiliensis. Plant Breed 124:86-92.

115. Sargent DJ, Hadonou AM, Simpson DW (2003) Development and characterization of polymorphic microsatellite markers from *Fragaria viridis*, a wild diploid strawberry. Mol Ecol Notes 3:550-553.

116. Sargent DJ, et al. (2007) Mapping A_1_ conferring resistance to the aphid Amphorophora ideai and dw (dwarfing habit) in red raspberry (Rubus ideaus L.) using AFLP and microsatellite markers. BMC Plant Biology 7:15 doi:10.1186/1471-2229-7-15.

117. Scalfi M, et al. (2004) A RAPD, AFLP, and SSR linkage map, and QTL analysis in European beech (*Fagus sylvatica* L.). Theor Appl Genet 108:433-441.

118. Schmidt AL, Scott L, Lowe AJ (2006) Isolation and characterization of microsatellite loci from *Macadamia*. Mol Ecol Notes 6:1060-1063.

119. Schwarz-Sommer Z, et al. (2003) A linkage map of an F2 hybrid population of *Antirrhinum majus* and *A. molle*. Genetics 163:699-710.

120. Sereno ML, Albuquerque PSB, Venconsky F, Figueira A (2006) Genetic diversity and natural population structure of cacao (*Theobroma cacao* L.) from the Brazilian Amazon evaluated by microsatellite markers. Cons Genet 7:13-24.

121. Sewell MM, Sherman BK, Neale DB (1999) A consensus map for Loblolly pine (*Pinus taeda* L.) I. Construction and intregration of individual linkage maps from two outbred three-generation pedigrees. Genetics 151:321-330.

122. Shokeen B, Kumar Sethy N, Bhatia S (2007) Isolation and characterization of microsatellite markers for analysis of molecular variation in the medicinal plant Madagascar periwinkle (*Catharanthus roseus* (L.) G. Don.). Plant Sci 172:441-451.

123. Silva Ritschel P, et al. (2004) Development of microsatellite markers from an enriched genomic library for genetic analysis of melon (*Cucumis melo* L.) BMC Plant Biol 4:9 doi:10.1186/1471-2229-4-9

124. Somta P, et al. (2008) New microsatellite markers isolated from mungbean (*Vigna radiata* (L.) Wilczek). Mol Ecol Res. In press. doi: 10.1111/j.1755-0998.2008.02219.x

125. Spada A, et al. (1998) A genetic map of *Asparagus officinalis* based on integrated RFLP, RAPD, and AFLP molecular markers. Theor Appl Genet 97:1083-1089.

126. Studer B, Widmer F, Enkerli J, Kölliker R (2006) Development of novel microsatellite markers for the grassland species *Lolium multiflorum*, *Lolium perenne*, and *Festuca pratensis*. Mol Ecol Notes 6:1108-1110.

127. Takahashi T, Tani N, Taira H, Tsumura Y (2005) Microsatellite markers reveal high allelic variation in natural populations of *Criptomeria japonica* near refugial areas of the last glacial period. J Plant Res 118:83-90.

128. Tani N, et al. (2003) A consensus linkage map for sugi (*Criptomeria japonica*) from two pedigrees, based on microsatellites and expressed tags. Genetics 165:1551-1568.

130. Tanksley SD, et al. (1992) High density molecular linkage maps of the tomato and potato genomes. Genetics 132:1141-1160.

131. Thamarus KA, et al. (2002) A genetic linkage map for *Eucalyptus globulus* with candidate loci for wood, fibre and floral traits. Theor Appl Genet 104:379-387.

132. Thiel T, Michalek W, Varshney R, Graner A (2003) Exploiting EST databases for the development and characterization of gene-derived SSR-markers in barley (*Hordeum vulgare* L.). Theor Appl Genet 106:411-422.

133. Tian-Ming H, et al. (2007) Using SSR markers to determine the population genetic structure of wild apricot (*Prunus armeniaca* L.) in the Ily Valley of West China. Genet Res Crop Evol 54:563-572.

134. Troggio M, et al. (2007) A dense single-nucleotide polymorphism-based genetic linkage map of grapevine (*Vitis vinifera* L.) anchoring pinot noir bacterial artificial chromosome contigs. Genetics 176:2637-2650.

135. Tsarouhas V, Gullberg U, Largercrantz U (2003) Mapping of quantitative trait loci controlling timing of bud flush in *Salix*. Hereditas 138:172-178.

136. Van Stallen N, Vanderbussche B, Verdoodt V, De Proft M (2003) Construction of a genetic linkage map of witloof (*Chicorium intybus* L. var. *foliosum* Hegi). Plant Breed 126:521-525.

137. Van Treuren R, et al. (1997) Evolution of microsatellites in *Arabis petraea* and *Arabis lyrata*, outcrossing relatives of *Arabidopsis thaliana*. Mol Biol Evol 14: 220-229.

138. Vanhala TK, Stam P (2006) Quantative trait loci for seed dormancy in wild barley (*Hordeum spontaneum* C. Koch). Genet Res Crop Evol 53:1013-1019.

139. Van Ooijen JW, et al. (1994) An RFLP linkage map of *Lycopersicon peruvianum*. Theor Appl Genet 89:1007-1013.

140. Venkateswarlu, M et al. (2006) A first linkage genetic map of mulberry (Morus spp.) using RADP, ISSR, and SSR markers nad pseudotestcross mapping strategy. Tree Genet Genomes 3:15-24.

141. Vivek BS, Simon PW (1999) Linkage relationships among molecular markers and storage root traits of carrot (*Daucus carota* L. ssp. *sativus*) Theor Appl Genet 99:58-64.

142. Wang XW, Kaga A, Tomooka N, Vaughan DA (2004) The development of SSR markers by a new method in plants and their application to gene flow studies in azugi [*Vigna angularis* (Willd.) Ohwi & Ohashi]. Theor Appl Genet 109:352-360.

143. Wang Y, et al. (2005) Inheritance of SSR markers and their use for genetic diversity analysis in wild and domesticated pawpaw [Asimina triloba (L.) Dunal]. J Amer Soc Hort Sci 130:561-568.

144. Westerbergh A, Doebley J (2002) Morphological traits defining species differences in wild relatives of maize are controlled by multiple quantitative trait loci. Evolution 56:273-283.

145. Yasui Y, Wag Y, Ohnishi O, Campbell CG (2004) Amplified fragment length polymorphism linkage analysis of common buckwheat (*Fagopyrum esculentum*) and its wild self-pollinated relative *Fagopyrum homotropicum*. Genome 47:345-351.

146. Zhao W, et al. (2005) Isolation and characterization of microsatellite loci from the mulberry, *Morus* L. Plant Sci 168:519-525.

147. Zwettler D, Vieira CP, Schöltterer C (2002) Polymorphic microsatellites in *Antirrhinum* (Scropulariaceae), a genus with low levels of nuclear sequence variation. J Hered 93:217-221.
