# Supplementary material for: The contribution of recombination to heterozygosity differs among plant evolutionary lineages and life-forms
Source: BMC Evol Biol. 2010 Jan 25;10:22. doi: 10.1186/1471-2148-10-22 (PMC2826329; doi:10.1186/1471-2148-10-22)
Supplement: Additional file 2 — Comparison of estimates of nucleotide diversity and recombination rates across different types of wild plant life-forms based on nuclear gene DNA sequences from population studies. [file 1471-2148-10-22-S2.DOC]

**Additional file 2:** Comparison of estimates of nucleotide diversity and recombination rates across different types of wild plant life-forms based on nuclear gene DNA sequences from population studies.

| Species | Genes^a^ | *n*^b^ | *l*^c^ | *S*^d^ | *θ*_π_ ^e^ | *hap*^f^ | *Rm*^g^ | *Rm*/*S*_p_ | Composite-likelihood | | |  | Summary statistics | | Source of  DNA sequences ^m^ |
| --- | --- | --- | --- | --- | --- | --- | --- | --- | --- | --- | --- | --- | --- | --- | --- |
|  |  |  |  |  |  |  |  |  | *S*_p_^h^ | *ρ*_MC_ ^i^ | *ρ*_MC_/*θ*_MC_ ^j^ |  | *ρ*_T05_ ^k^ | *ρ* _T05_/*θ* _T05_ ^l^ |  |
| Trees |  |  |  |  |  |  |  |  |  |  |  |  |  |  |  |
| Conifers |  |  |  |  |  |  |  |  |  |  |  |  |  |  |  |
| *Cryptomeria japonica* | 3 | 48 | 1702 | 23 | 4.017 | 12 | 0.33 | 0.02 | 19 | 0.803 | 0.319 |  | 1.026 | 0.313  (0.128-0.867) | 62 |
| *Pseudotsuga menziesii* | 5 | 26 | 1547 | 37 | 5.886 | 14 | 3.20 | 0.12 | 26 | 4.434 | 1.820 |  | 4.375 | 0.749  (0.539-1.055) | 63 |
| *Pinus taeda* | 8 | 32 | 1369 | 25 | 4.764 | 12 | 1.62 | 0.09 | 18 | 3.164 | 0.983 |  | 3.276 | 0.753  (0.396-1.172) | 34; 64 |
| *Pinus pinaster* | 4 | 33 | 1057 | 18 | 5.448 | 8 | 1.50 | 0.11 | 14 | 1.629 | 0.677 |  | 2.736 | 0.727  (0.194-1.518) | Own unpub. data |
| Broadleaves |  |  |  |  |  |  |  |  |  |  |  |  |  |  |  |
| *Quercus crispula* | 3 | 170 | 1167 | 76 | 6.857 | 62 | 12.00 | 0.32 | 37 | na | na |  | na | na | 61 ^n^ |
| *Populus tremula* | 8 | 25 | 1612 | 64 | 8.431 | 22 | 14.13 | 0.35 | 40 | 42.319 | 6.791 |  | 93.476 | 10.875  (8.737-13.478) | 65, 66, 67 |
| Other plant life-forms |  |  |  |  |  |  |  |  |  |  |  |  |  |  |  |
| *Solanum peruvianum* | 6 | 23 | 1260 | 69 | 11.753 | 17 | 8.33 | 0.24 | 35 | 27.477 | 4.244 |  | 38.196 | 3.270  (1.950-5.019) | 68 |
| *Helianthus annuus* (wild) | 4 | 32 | 1118 | 39 | 6.272 | 16 | 6.00 | 0.19 | 32 | 13.283 | 2.787 |  | 24.770 | 4.291  (2.446-8.142) | 69 |
| *Zea mays* ssp. *parviglumis* | 5 | 26 | 1198 | 75 | 11.649 | 23 | 8.80 | 0.21 | 42 | 60.882 | 6.574 |  | 59.749 | 4.915  (3.225-7.741) | 70 |
| *Hordeum spontaneum* | 7 | 25 | 1333 | na | 6.544 | 16 | 3.43 | 0.15 | 23 | 9.640 | 1.479 |  | 9.020 | 1.282  (0.563-4.443) | 3 ^o^ |

Nomenclature and data analyses as in ref. [3]:

^a^ Number of genes.

^b^ Number of sampled chromosomes (≥20 per gene).

^c^ Average aligned sequence length per gene in base pairs (≥800 bp per gene).

^d^ Number of segregating sites (≥10 per gene, at least 6 parsimony informative).

^e^ Population-scaled mutation rate (*θ* ; per site × 10^-3^) based on the average number of nucleotide differences.

^f^ Number of haplotypes.

^g^ Minimum number of recombination events [56].

^h^ Number of parsimony informative segregating sites with two alleles.

^i^ Composite-likelihood estimate of the population-scaled recombination rate (*ρ* ; per site × 10^-3^) [58; 59].

^j^ Recombination to mutation ratio, calculated by using *θ* and *ρ* estimates from the composite-likelihood method as implemented in LDhat [58; 59], excepting wild barley for which the original estimate (computed with *maxhap*, [58]) is given [See ref. 3].

^k^ Estimate of *ρ* (per site) × 10^-3^ based on summary statistics [60].

^l^ Co-estimated *θ* /*ρ* ratio based on summary statistics [60]. 95% confidence intervals between brackets.

^m^ Original publications from which sequences were obtained (see Material and Methods and references below).

^n^ Estimates for *Quercus crispula* were taken from the original publication (61), as DNA sequences are not publicly available.

^o^ Averages for wild barley were computed based on the per locus estimates provided in the original publication, but only including the loci with *n*≥20, *l*≥800 bp and *S*_p_≥6, and which did not show population structure [see ref. 3].

na = not available.

**References**

3. Morrell PL, Toleno DM, Lundy KE, Clegg MT: **Estimating the contribution of mutation, recombination and gene conversion in the generation of haplotypic diversity**. *Genetics* 2006; **173:**1705-1723.

34. Brown GR, Gill GP, Kuntz RJ, Langley CH, Neale DB: **Nucleotide diversity and linkage disequilibrium in loblolly pine**. *Proc. Natl. Acad. Sci. USA* 2004; **101:**15255-15260.

62. Kado T, Yoshimaru H, Tsumura Y, Tachida H: **DNA variation in a conifer, *Cryptomeria japonica* (Cupressaceae sensu lato)**. *Genetics* 2003; **164:**1547-1559.

63. Krutovsky KV, Neale DB: **Nucleotide diversity and linkage disequilibrium in cold hardiness and wood quality related candidate genes in Douglas-fir**. *Genetics* 2005; **171:**2029-2041.

64. González-Martínez SC, Ersoz E, Brown GR, Wheeler NC, Neale DB: **DNA sequence variation and selection of tag Single-Nucleotide Polymorphisms at candidate genes for drought-stress response in *Pinus taeda* L**. *Genetics* 2006; **172:**1915-1926.

65. Ingvarsson PK: **Nucleotide polymorphism and linkage disequilbrium within and among natural populations of European aspen (*Populus tremula* L., Salicaceae)**. *Genetics* 2005; **169:**945-953.

66. Ingvarsson PK, García MV, Hall D, Lúquez V, Jansson S: **Clinal variation in phyB2, a candidate gene for day-length induced growth cessation and bud set, across a latitudinal gradient in European aspen (*Populus tremula*)**. *Genetics* 2006; **172:**1845-1853.

67. Ingvarsson PK, García MV, Lúquez V, Hall D, Jansson S: **Nucleotide polymorphism and phenotypic associations within and around the phytochrome B2 locus in European aspen (*Populus tremula*, Salicaceae)**. *Genetics* 2008; **178:**2217-2226.

68. Arunyawat U, Stephan W, Städler T: **Using multilocus sequence data to assess population structure, natural selection, and linkage disequilibrium in wild tomatoes**. *Mol. Biol. Evol.* 2007; **24:**2310-2322.

69. Liu A, Burke JM: **Patterns of nucleotide diversity in wild and cultivated sunflower**. *Genetics* 2006; **173:**321-330.

70. Moeller DA, Tenaillon MI, Tiffin P: **Population structure and its effects on patterns of nucleotide polymorphism in teosinte (*Zea mays* ssp. *parviglumis*)**. *Genetics* 2007; **176:**1799-1809.
